# Concentrated Polymer Brush-Modified Magnetic Particles for a Diagnostic Immunoassay

**DOI:** 10.1021/acs.langmuir.5c04175

**Published:** 2025-11-25

**Authors:** Gabriel Tai Huynh, Jun Qiu, Edith van den Bosch, Tomohiko Yamazaki, Chiaki Yoshikawa

**Affiliations:** † Research Center for Macromolecules and Biomaterials, 52747 National Institute for Materials Science (NIMS), 1-2-1 Sengen, Tsukuba, Ibaraki 305-0047, Japan; ‡ DSM Ahead/TS, 6167 DR Geleen, The Netherlands; § Graduate School of Life Science, Hokkaido University, Kita 10, Nishi 8, Sapporo 060-0808, Japan

## Abstract

Nonspecific protein absorption is an ongoing problem in the development of highly sensitive magnetic particle (MP)-based diagnostic assays, whereby the MP surface undergoes biofouling, significantly reducing the limit of detection and sensitivity of the device. In this study, a bioinert, concentrated polymer brush (CPB) composed of poly­[poly­(ethylene oxide) methyl ether methacrylate] (PPEGMA) was employed to reduce the absorption of protein onto the MP surfaces; and an anti-human serum albumin antibody (Ab) was then immobilized onto the brush layer by click chemistry to demonstrate its application as an immunoassay platform. The amount of antibody grafted onto the ends of the brush coating was quantified by a BCA assay with a high grafting efficiency (∼0.80 antibody per chain). Furthermore, when using the antibody-conjugated CPB-coated MPs (MP-PPEGMA-Ab) as an immunoassay platform, we were able to determine the capture efficacy of human serum albumin (HSA) in both a buffered solution and diluted human serum by colorimetric analysis, and further confirmation was achieved via liquid chromatography/mass spectrometry, in which our MPs showed a high selectivity toward the targeted analyte. Lastly, we demonstrated that MP-PPEGMA-Ab was capable of detecting human serum albumin at a concentration as low as 6.4 ng mL^–1^, 30% more sensitive than the unmodified MP, demonstrating the impact of CPBs on diagnostic assays. Due to their high selectivity and sensitivity, our CPB-based MPs are expected to be applicable for a wide range of immunoassay applications by employing different bioinert polymers and biofunctional groups.

## Graphical abstract

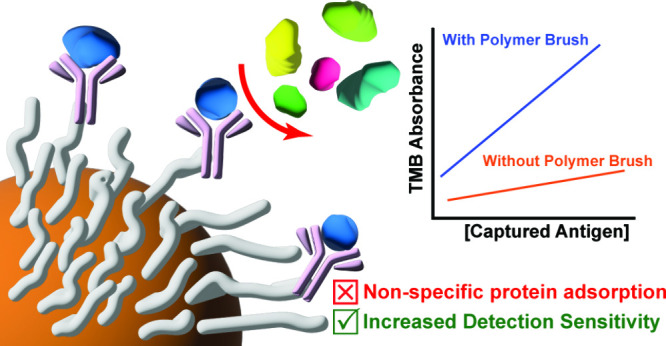

## 1 Introduction

Magnetic particles (MPs) have been used extensively in biomedical applications, as either a contrast agent for deep tissue imaging,^1–3^ cancer therapeutic (hyperthermia) agents,^4–6^ or drug carriers for cancer treatment or for selectively capturing and/or separating cells,^7^ proteins, and/or DNA from complex biological matrices. Compared to other purification techniques such as ionic precipitation, dialysis, and electrophoresis,^8–10^ MP separation requires minimal technical expertise, can be easily scaled, and does not require a large capital investment. Therefore, MPs have been used in multiple applications for selective capture and separation, such as capturing and separating tumor cells from blood,^11,12^ extracting antigens for viral detection,^11,13,14^ or concentrating low-abundance proteins in developing highly sensitive assays.^15^ These surfaces are typically modified with bioactive groups such as antibodies or peptides, which selectively bind to the target molecule, allowing for easy capture and subsequent extraction. Conversely, due to the complex nature of biological solutions, such as protein-rich serum, nonspecific protein adsorption and interactions would inevitably lead to poor binding and poor sensitivity and selectivity. Therefore, in order to enhance the selectivity and sensitivity of biosensors, bioinert coatings have been explored to prevent and minimize nonspecific protein absorption.

Polymer coatings of biocompatible hydrophilic polymers such poly­(ethylene) glycol (PEG) or zwitterionic polymers^16–18^ have been used to suppress nonspecific protein adsorption. Typically, these polymers are grafted onto the surface through physisorption^19,20^ or chemical conjugation of preformed polymer chains.^21^ However, the efficacy of these coatings can greatly vary for several reasons, such as the polymer chain length,^22,23^ grafting density^24,25^ and spacing,^26,27^ polymer orientation and conformation,^28,29^ and chemical stability^30^ all affecting their performance. Recently, surface-initiated atomic transfer radial polymerization (SI-ATRP) has been explored as a method for grafting polymers, such as PEG^31,32^ or zwitterionic^33–36^ polymers onto surfaces,^35^ producing well-defined polymer brush structures with a high grafting density and good long-term stability.^37^ Unlike conventional radial polymerization, the surface is modified with an alkyl halide group, whereby the distribution of the decapping and capping of the halogen initiator can be controlled, which allows for high-density polymer grafting. SI-ATRP has been successfully applied to silicon^38^ and gold surfaces,^39,40^ silica nanoparticles,^41,42^ and graphene oxide,^43^ where these surfaces were modified to minimize protein adsorption. The Tsujii group has extensively explored the use of high-density polymer brushes, otherwise known as concentrated polymer brushes (CPBs),^44^ for antifouling applications. They have reported that CPBs form unique high-extension structures when swollen in aqueous solvents, allowing them to have strong durability, long-term stability, and a super lubrication effect, giving rise to their antifouling properties. Moreover, we recently demonstrated the unique size-exclusion effect of CPBs by varying both the graft density and chemical composition of various polymers, while confirming that CPBs significantly suppressed protein adsorption and subsequent cell adhesions when directly compared against thin films and coatings with a semidilute polymer brush (SDPB) configuration of the same corresponding polymers.^38,42,45^ Such bioinert CPBs are expected to be useful base coating to enhance selective binding of the functional groups on MPs (Scheme 1).

**Scheme 1:**
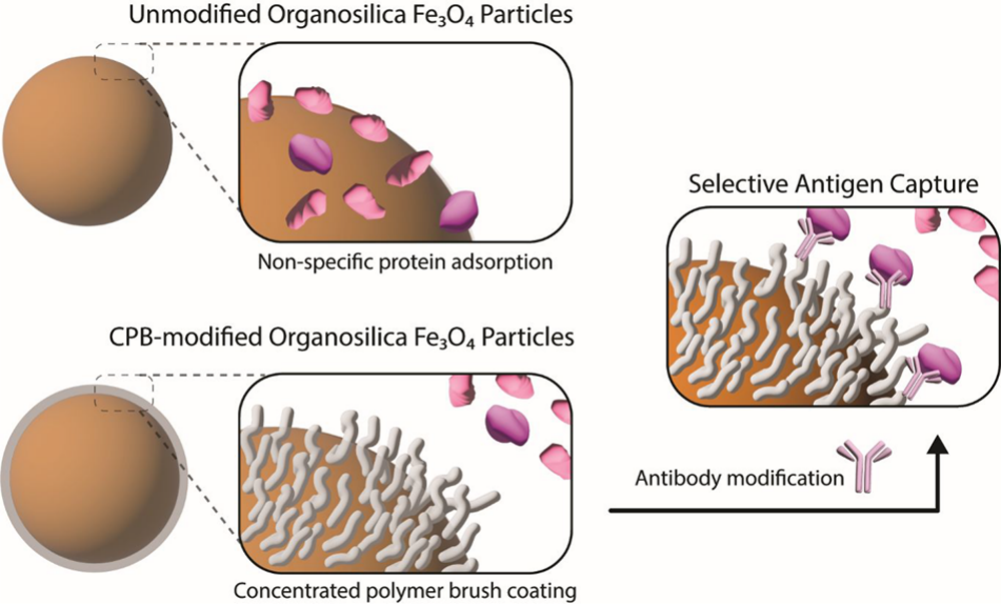
Graphical Representation of the Scope of the Study^a^

a Organosilica-modified iron oxide (Fe_3_O_4_)-based magnetic particles (MPs) were modified with a concentrated polymer brush (CPB) coating to prevent nonspecific protein adsorption and subsequently modified with an antibody for selective antigen capture.

In this work, we developed CPB-modified MPs that can selectively capture and concentrate a target protein. While CPB-modified magnetic particles have been seen countless times in the literature,^46–48^ its use as an antifouling coating for improving immunoassays, to the best of our knowledge, has yet to be realized. By using commercially available hydroxyl-functionalized organosilica-coated MPs, we first grafted poly­[poly­(ethylene glycol) methyl ether methacrylate] (PPEGMA), which has been reported as one of the biocompatible polymers, onto the surface using SI-ATRP, which we previously reported to have good stability and functional lifetimes when previously grafted onto silica particles,^37,49^ or when used as a preventative coating within *in vivo* settings.^42^ Subsequently, an antibody for targeted antigen capture was immobilized at the chain end of grafted PPEGMA by click chemistry. Here, the targeted analyte was chosen to be human serum albumin (HSA) as a representative target due to its high abundance in serum. Finally, we confirmed that the presence of CPB on MPs improved the sensitivity for immunoassays by indirect and direct analysis (Scheme 2). Following incubation of the MPs with human serum and plasma, (1) unreacted HSA in serum and plasma was quantified with an enzyme-linked immunosorbent assay (ELISA) (indirect analysis) and (2) HSA bound on the MPs was quantified by direct enzyme–substrate binding, namely, adding enzyme and substrate to the solution of MPs (direct analysis). The direct analysis showed that MPs with CPB had a lower limit of detection of 6.4 ng mL^–1^, approximately 30% more sensitive than MPs without the CPB coating.

**Scheme 2:**
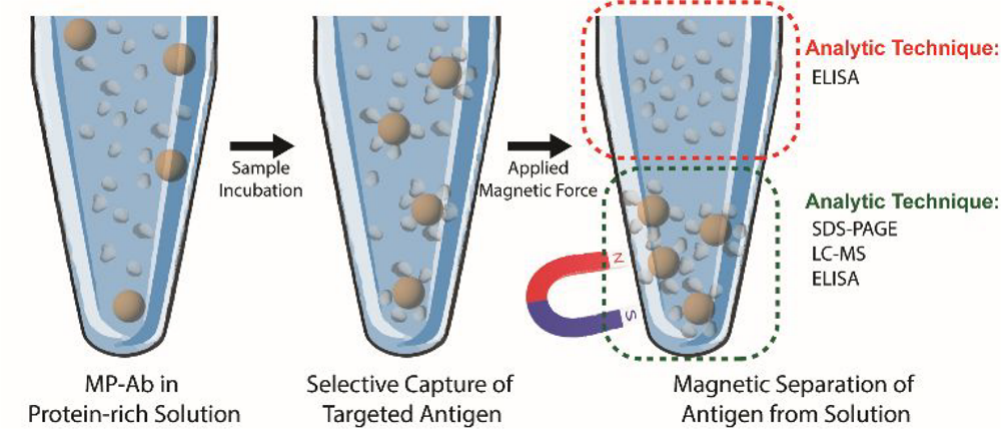
Selective Capture and Subsequent Analysis of MP-PPEGMA-Ab toward the Targeted Analyte^a^

a Here, MP-PPEGMA-Ab is incubated in a protein solution, where magnetic separation is applied, separating both captured and noncaptured proteins in solution.

As a proof of concept, in this work, we used PPEGMA as a bioinert polymer and HSA as a target antigen. Since our MP coated with a CPB layer offers a wide range of design possibilities, by varying the type of polymer and biofunctional groups, this work provides a stepping stone for developing new and highly sensitive diagnostic tools for various diseases and illustrates the importance of bioinert coatings for immunoassays.

## 2 Materials and Methods

### 2.1 Materials

Ethyl-2-bromoisobutyrate (EBIB, 98.0%, Tokyo Chemical Industry Co., Ltd. (TCI), Japan), copper­(I) bromide (Cu­(I)­Br, 99.9%, Wako Pure Chemical Industries, Ltd., Japan), 2,2′-bipyridyl (Bpy, 99.5%, Nacalai tesque, Japan), and *N*,*N*,*N*′,*N*″,*N*″-pentamethyldiethylenetriamine (PMDETA, 99.0%, Tokyo Chemical Industry Co., Ltd. (TCI), Japan) were used as received. {[(2-Bromo-2-methylpropionyl)­oxy]­propyl} triethoxysilane (BPE) was synthesized according to the literature.^41^ Poly­(ethylene oxide) methyl ether methacrylate (average *M*_n_ of ∼475) (PEGMA Sigma-Aldrich, Osaka, Japan) was purified by being passed through neutral alumina. Hydroxyl-functionalized organosilica-coated iron oxide magnetic particles (MPs) (SiMAG-Hydroxyl, diameter of 500 nm) were purchased from chemicell GmbH (Berlin, Germany).

### 2.2 Immobilization of BPE on MPs

SiMAG-hydroxyl particles (MPs) were washed with absolute ethanol (EtOH) three times. Then the MPs (100 mg), aqueous ammonia (28% (w/w) in water, 1.52 g), and EtOH (11.00 mL) were placed in a flask. BPE (0.22 g) in EtOH (2 mL) was then added to the flask, and its contents were mixed at room temperature for 18 h. After the reaction, BPE-modified MPs were washed with absolute ethanol three times. Subsequently, the MPs were washed with methanol (MeOH) twice, and the concentration of MPs in MeOH was adjusted to 5.0% (w/v). The BPE-modified MPs (MP-Br) in MeOH were stored at 4 °C until use.

### 2.3 SI-ATRP of PPEGMA onto MPs

Concentrated PPEGMA brushes were grafted onto MPs by surface-initiated atom transfer radial polymerization (SI-ATRP). Briefly, MP-Br (25 mg) was dispersed in a N_2_-purged MeOH solution (6.60 g) of PEGMA (6.60 g, 13.94 mmol), Cu­(I)Br (10.0 mg, 0.070 mmol), Bpy (21.78 mg, 0.139 mmol), and free initiator EBIB (13.59 mg, 0.070 mmol), and the solution was stirred at 30 °C for 3.5 h. After polymerization, the modified particles were washed in methanol, and the concentration of MP-PPEGMA-Br was adjusted to 10 mg mL^–1^ in MeOH and stored at 4 °C until use. After polymerization, the number-averaged molecular weight (*M*_n_) and the weight-averaged molecular weight (*M*_w_) of free polymers were determined by gel permeation chromatography (GPC) using *N,N*-dimethylformamide (DMF) with 10 mM lithium chloride (LiCl), with poly­(methyl methacrylate) (PMMA) calibration standards. Free polymer conversion was determined by proton nuclear magnetic resonance (^1^H NMR) measurements in deuterated chloroform. The theoretical *M*_n_ (*M*_n,conv_) was calculated by

1 $$M_{n,\mathrm{conv}}={\left[M\right]}_{0}/{\left[\mathrm{EBIB}\right]}_{0}\times \mathrm{MW}\times C$$

where MW is the molecular weight of the PEGMA monomer and *C* is the monomer conversion (per 100%) determined by ^1^H NMR.

The amount of grafted PPEGMA (weight loss) was estimated by thermal degradation–differential thermal analysis (TG-DTA, TG8120, RIGAKU Co., Ltd., Tokyo, Japan). Grafting amount σ (number of chains per square nanometer) was estimated by

2 $$\sigma ={AN}_{A}/\left(M_{n,\mathrm{conv}}S\right)$$

where *A* is the graft amount (grams per gram) and *S* is the particle surface area (square nanometers per gram).

The dimensionless graft density (σ*) was calculated by

3 $$\sigma^{*}=a^{2}\sigma$$

where *a* ^2^ is the cross-sectional area per monomer.

### 2.4 Terminal Azidation of PPEGMA Brushes

MP-PPEGMA-Br (20 mg) particles were suspended in 1.00 g of DMF before being mixed with a solution of DMF (1.00 g) containing sodium azide (NaN_3_, 0.267 mg, 4.1 × 10^–3^ mol). The reaction mixture was then mixed at 50 °C for 18 h. After the reaction, MP-PPEGMA-N_3_ was thoroughly washed with DMF before being adjusted to a concentration of 10 mg of MP-PPEGMA-N_3_ in 1 g of DMF and stored at 4 °C until use.

### 2.5 Azide–Alkyne Click Chemistry for PPEGMA Functionalization

*N*-(4-Pentynoyloxy) succinimide (30.7 mg), Cu­(I)Br (75 mg), PMDETA (90.6 mg), and DMF (7.50 g) were placed in a Schlenk tube, and then, the MP-PEGMA-N_3_ in DMF (75 mg in 75 g of DMF) was added to the tube. The mixture was vigorously mixed at room temperature for 18 h. After the click reaction, the resulting MP-PPEGMA-NHS was washed with MeOH. The MP-PPEGMA-NHS was then dispersed in dimethyl sulfoxide (DMSO) (10 mg of MP-PEGMA-NHS in 1 g of DMSO) and kept at 4 °C until use.

### 2.6 Fixation and Quantification of the Antibody on MPs

MP-PPEGMA-NHS and pristine MPs were first suspended in 25 mM MES-NaOH buffer (10 mg mL^–1^), before the addition of 400 μL of the goat anti-human albumin antibody (HSA antibody, 1 mg mL^–1^, Bethyl Laboratories, Inc., Montgomery, TX). The solution was then mixed at 4 °C for 1 h. Following the reaction, the samples were centrifuged at 15 000 rpm for 5 min, where the amount of unbound antibodies present within the supernatant was quantified through a microbicinchoninic acid (micro-BCA) assay (Thermo Fisher Scientific, Waltham, MA). Subsequently, the MP-antibody particles were washed thoroughly with fresh HEPES buffer (10 mM HEPES-NaOH (pH 7.9)), 50 mM potassium chloride (KCl), 1 mM ethylenediaminetetraacetic acid (EDTA), and 10% (v/v) glycerin. The modified particles were adjusted to a concentration of 10 mg mL^–1^ and stored at 4 °C.

### 2.7 Human Serum and Plasma Protein Adsorption Testing

MP-PPEGMA-Ab particles (5.0 mg) was first suspended in 100 μL of a 1× phosphate-buffered saline (PBS) solution. Then, 900 μL of human reference serum (RS10-110-4, Bethyl Laboratories, Inc.) or human whole plasma (human EDTA-2Na plasma, single donor) (KAC Co., Ltd., Kyoto, Japan) was added to the MP solution. The mixture was then incubated at 37 °C for 1 h, before being washed five times with 1 mL of 1× PBS to remove all unbound proteins. After being washed, the MPs were centrifuged at 15 000 rpm for 4 min, and the supernatant was discarded. The proteins bound to the surface were quantified by first adding 500 μL of a 5.0% (w/v) sodium dodecyl sulfate (SDS) solution to the captured magnetic particles. The solution was mixed for 1 h, allowing all of the bound proteins to detach from the surface. Then, the supernatant was collected by centrifugation, where the amount of human albumin was quantified by an ELISA (E80-129, Human Albumin ELISA Quantitation Set, Bethyl Laboratories, Inc.).

### 2.8 Gel Electrophoresis

Following adsorption of the protein to the magnetic particles, the concentration of the particle suspension was adjusted with 1× PBS to a final concentration of ∼0.25 mg μL^–1^. In a typical run, 10 μL of the solution was loaded into a polyacrylamide gel (NuPAGE 4–12% Bis-Tris gel, Thermo Fisher). Gel electrophoresis was conducted in accordance with the manufacturer’s provided protocol. Afterward, the bands were visualized by silver staining (AE-1360 EzStain Silver, ATTO, Japan) and imaged with a scanner (CanonScan8800F, Canon, Japan).

### 2.9 Direct Measurement of Albumin Captured on the MPs

Prior to the experiment, the amount of albumin presented in human whole plasma (human EDTA-2Na plasma, single donor) (KAC Co., Ltd.) was determined to be 65 mg mL^–1^ by an ELISA. Following this, human whole plasma was diluted in the sample diluent solution (50 mM Tris-HCl, 0.14 M sodium chloride (NaCl), 1.0% (w/v) bovine serum albumin (BSA), and 0.025% (v/v) Tween 20 (pH 8.0), Bethyl Laboratories, Inc.), as described by the manufacturer’s protocol. The sample was diluted until the final concentration of human albumin present was 1.00 mg mL^–1^ (1000 000 ng mL^–1^).

Then, 5.0 mg of either MP-PPEGMA-Ab or pristine MP-Ab was suspended in 100 μL of 1× PBS, before the addition of 900 μL of the diluted human whole plasma solution. The solution was mixed and incubated at 37 °C for 1 h. Afterward, the MPs was washed five times with 1 mL of 1× PBS to remove any unbound proteins, before being resuspended in 1.0 mL of 1× PBS.

Then, 500 μL of the resuspended particle solution was mixed with 50 μL of a solution containing a horseradish peroxide (HRP)-conjugated anti-HSA detection Ab solution (Bethyl Laboratories, Inc.). The solution was mixed for 1 h at room temperature, before being centrifuged and washed 10 times in 1.0 mL of an ELISA washing buffer solution (Bethyl Laboratories, Inc.). Subsequently, 50 μL of a 3,3′,5,5′-tetramethylbenzidine (TMB) substrate solution was added to the particle suspension and mixed for 10 min, before 25 μL of a stop solution (Bethyl Laboratories, Inc.) was added to the MP suspension. The amount of HSA present on the surface of the magnetic particles was then quantified by the absorbance at 450 nm, where 75 μL of the particle solution was added to a 96-well plate.

## 3 Results and Discussion

### 3.1 Synthesis and Characterization of CPB-Modified MPs

The surface modifications of the hydroxyl-functionalized organosilica-coated MP are shown in Figure 1. Organosilica-modified magnetic particles were selected due to the ease of the subsequent surface modification and good colloidal stability.^50^ In order to first graft our polymers onto the organosilica-MP surface, we began by modifying the surface with BPE, a fixed initiator for ATRP (Figure 1, I). Next, PEGMA was grafted onto the surface through SI-ATRP (Figure 1, II). The characterization of the PPEGMA coating on the magnetic particles can be found in Table S2. Here, the number-weighted average molecular weight (*M*_n_) and polydispersity (*M*_w_/*M*_n_) were determined from the free polymer formed during the reaction. It has been widely established that both the *M*_n_ and the *M*_w_/*M*_n_ of the free polymer are highly representative of both the *M*_n_ and the *M*_w_/*M*_n_ of the grafted polymers formed via SI-ATRP,^51,52^ especially then compared and validated against high-resolution surface characterization techniques, such as X-ray photoelectron spectroscopy,^53^ atomic force microscopy,^41,54^ and transmission electron microscopy.^41,55^ Subsequently, we calculated the grafting density (σ) and its dimensionless counterpart (σ*) to confirm that the coating on our particles consisted of CPBs (σ* > 0.1) (Table S2).

**Figure 1:**
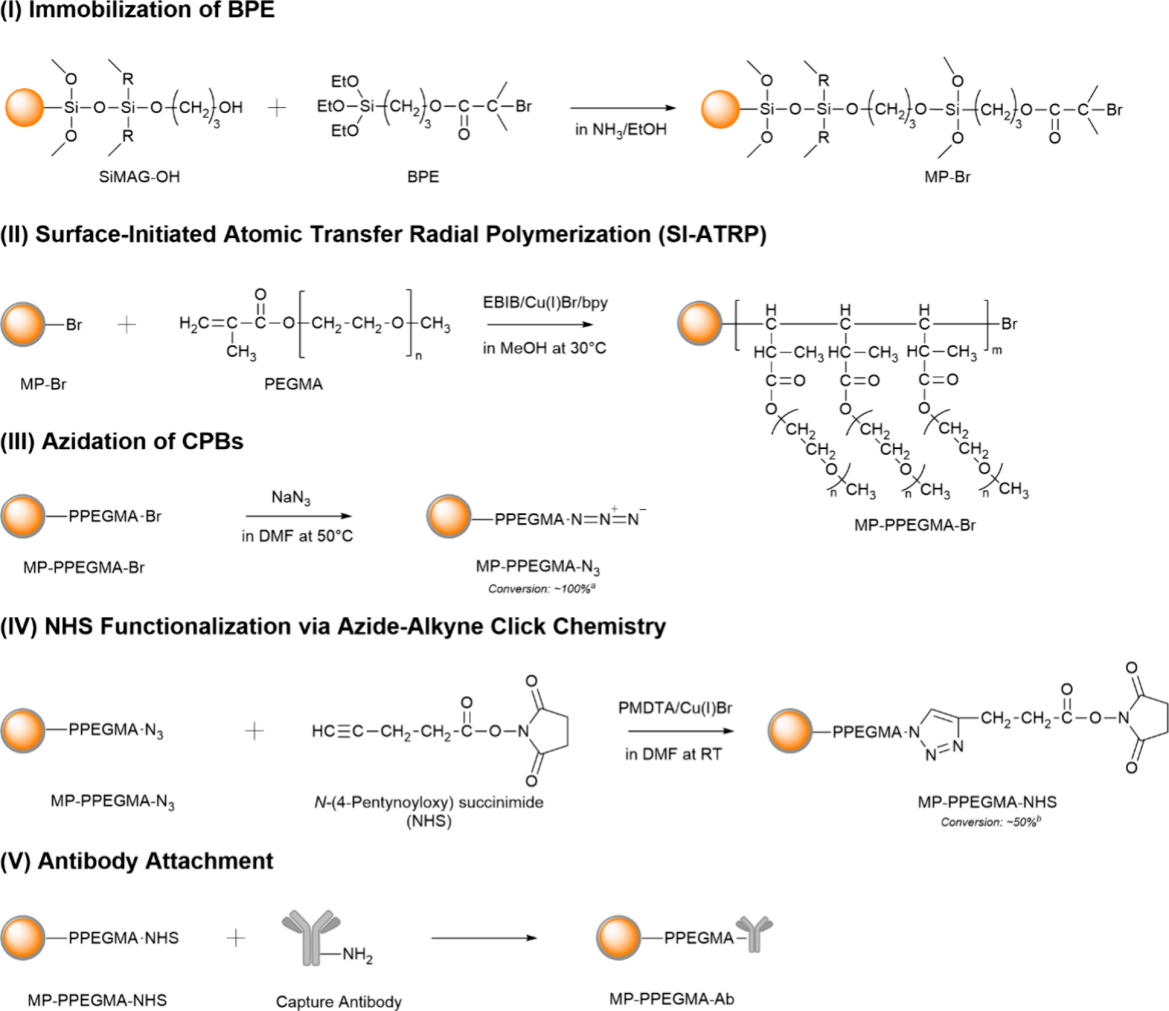
Synthesis of MP-PEGMA-Ab, where we surface modified commercially available hydroxyl-functionalized organosilica magnetic microparticles with (I) a bromide initiator, (II) an antifouling coating, (III) an azide terminating group, (IV) a NHS terminating group, and (V) an antibody. ^*a*^Azidation conversion was confirmed via ^1^H NMR (Figure S3b), and ^*b*^azide–alkyne click chemistry conversion was confirmed via ^1^H NMR (Figure S3c). The structure of SiMAG-OH was provided by the commercial supplier.

A schematic representation of the classification of the brush-like structure is shown in Scheme 3, where the distance between the anchor site (*d*) and the distance between the elongated polymer chains (*D*) are determining factors with respect to whether a polymeric coating is classified as CPB or SDPB.^51^ If the distance between two attachment points is in parity with the distance between neighboring polymer chains (*D* ≈ *d*), a repulsion barrier effect occurs in which proteins are unable to directly adsorb to the surface. Conversely, if the distance between the polymer chains is larger than the distance between neighboring anchor sites (*D* > *d*), proteins can diffuse through the brush-like structure, with smaller proteins being able to foul the surface.

**Scheme 3:**
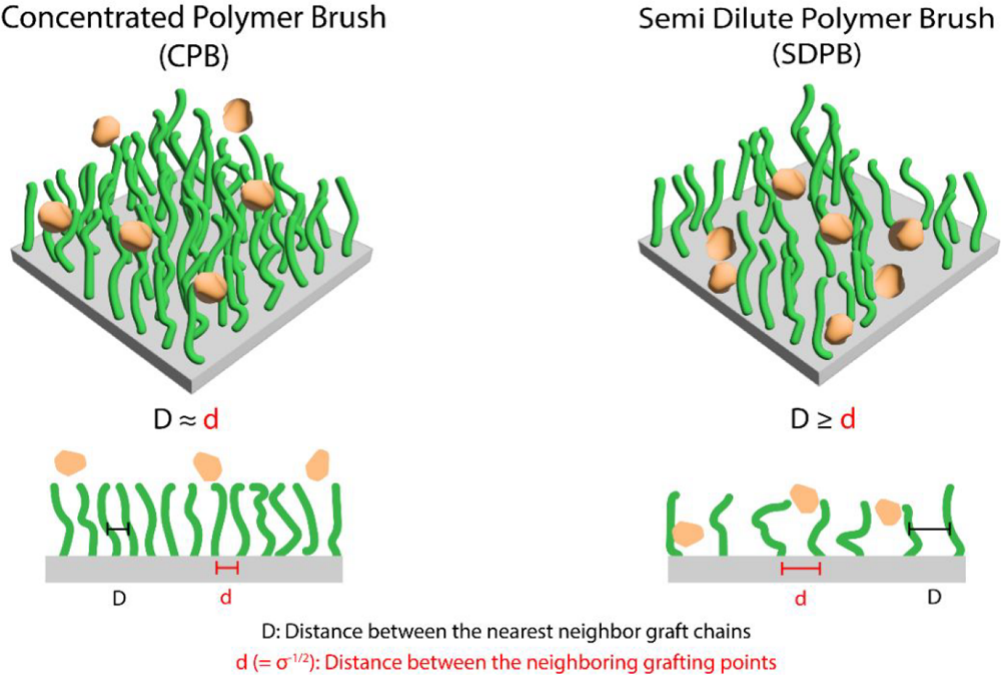
Schematic Representation of the Difference between Concentrated Polymer Brush (CPB) Structures and Semidilute Polymer Brush (SDPB) Structures When in a Well-Dispersed Solvent

Previously, we reported that CPBs grafted on both silicon wafers and silica particles exhibit excellent low biofouling capacities when compared against their corresponding SDPB counterparts,^42^ suggesting that our coatings would exhibit similar behavior. To validate our hypothesis, we compared both SDPB- and CPB-modified particles by incubating them in undiluted fetal bovine serum (FBS) for one hour before performing gel electrophoresis. As shown in Figure S1, particles modified with a SDPB coating (σ = 0.009 chain nm^–2^, and σ* *=* 0.03) showed evidence of biofouling (lane 3), whereby particles with a CPB coating (σ = 0.05 chain nm^–2^, and σ* *=* 0.16 (lane 2); σ = 0.58 chain nm^–2^, and σ* *=* 1.0 (lane 4)) showed no evidence of biofouling, conferring the antifouling effect from CPB coatings. It should be noted that while there was batch-to-batch variation between each particle set (Tables S2 and S3), mainly differences in the number-weighted average molecular weight values. However, as the definition of CPB and SDPB is dependent on the dimensionless grafting density value (σ*), the observed differences between *M*_n_ values did not affect the overall performance of the coating (Figure S1).

Next, to functionalize the particle surface with an antibody, we modified the terminal bromide group along the PPEGMA brushes with sodium azide, where the nucleophilic substitution (SN_2_) reaction resulted in the polymer brush containing an azide terminal moiety^56^ (Figure 1, III). The azide group was confirmed through FTIR analysis, with the latter showing a slight absorption peak around ∼2100 cm^–1^ (Figure S2). Furthermore, ^1^H NMR analysis on the free polymer chain further confirmed that the substitution reaction was approximately 100% (Figure S3). Previous studies by Sakakibara et al. similarly reported that the bromine at the chain end of concentrated PPEGMA brushes grafted on a silicon wafer could be converted into an azide group with approximately 100% efficiency, further validating our observations.^57^ Therefore, we considered that the azidation reaction would also proceed on our grafted polymers on the MPs.

Then, azide–alkyne click chemistry^58^ between *N*-(4-pentynoyloxy) succinimide was used to further modify the PPEGMA brush to have a terminal hydroxy-succinimide group, which readily reacts with primary amines on the captured antibody (Figure 1, IV). ^1^H NMR analysis of the free polymer revealed that most terminal azides reacted with alkyne hydroxy succinimide (Figure S3). Finally, we immobilized an anti-human albumin antibody (Ab) on the brush surface using the *N*-hydroxysuccinimide (NHS) group at the chain end (Figure 1, V). The amount of Ab on the MP surface was quantified by the BCA assay. The molar ratio of the Ab to grafted polymer chains was estimated to be between 0.73 and 0.86, signifying that a majority of the chains had been successfully modified (Table S4).

### 3.2 Identification of Adsorbed Proteins on MP-PPEGMA-Ab

To show the capabilities of our particles for specific analyte targeting, we used human serum albumin as a model target. HSA is one of the most abundant proteins present in serum, but low levels of albumin (hypoalbuminemia) have been associated with different diseases such as cardiovascular disease^59,60^ or kidney/renal disease.^61^ To capture HSA from human serum, we used an anti-HSA antibody (Ab) and subsequently attached it to our magnetic particles. Sodium dodecyl sulfate–polyacrylamide gel electrophoresis (SDS–PAGE) was performed to confirm the capture of the protein, where proteins are separated based on their relative size under an applied voltage (Figure S4). As HSA has a well-known size (67 kDa), we could easily identify the presence of the protein by gel electrophoresis. For experimental controls, we ran both anti-HSA Ab at a concentration of 1.0 mg mL^–1^ and MP-PPEGMA-Ab (Figure S4, lanes 2 and 3) and found that there was a noticeable band visible at ∼55 kDa, likely due to the light chains from the Ab complex. Similarly, by running human reference serum (Figure S4, lane 5), we saw multiple bands, which indicated the complex protein matrix of human serum. Subsequent gel digestion and LC-MS on MP-PPEGMA-Ab confirmed the presence of Ig λ chain V-III (Table 1, region 5), further validing the presence of anti-HSA on the surface of the particles. Interestingly, the top three scoring proteins tagged from the MP-PPEGMA-Ab (Table 1, region 5) were all associated with actin and cardiac cells^62–64^ (Shroom3, actin, aortic smooth muscle, and actin, α cardiac muscle), which can be found in human blood and serum.^65^ As the antibody solution (Table 1, regions 3 and 4) showed the presence of serum, it is likely that the appearance of these actin-related proteins would have been associated with it. Moreover, hemoglobin, fetal subunit β, and α-1-antiproteinase were present in the top scoring proteins within the protein solution. With these proteins being associated with blood,^66^ serum, and the immune response,^67,68^ the presence of these proteins was expected. Similarly, as actin plays a key role in the adhesion of cell to surfaces,^69^ it is unsurprising that these proteins would be present, likely competing against terminal NHS polymer chains.

**Table 1:** Top Three Scoring Proteins Found following Gel Digestion and LC-MS^a^

gel region	top scoring protein	second top scoring protein	third top scoring protein
3	serum albumin	α-1-antiproteinase	hemoglobin fetal subunit β
4	hemoglobin fetal subunit β	serum albumin	–
5^a^	shroom3	actin, aortic smooth muscle	actin, α cardiac muscle
11, 16, 20, 24, and 27	serum albumin	–	–
12	serum albumin	transient receptor potential cation channel subfamily V member 5	protein shroom
17, 21, 25, and 28	protein shroom	serum albumin	

a The corresponding gel can be found in Figure S5.

b The Ig λ chain V-III region LOI was present, identified as the 12th top scoring protein.

Similarly, in regions in which MP-PPEGMA-Ab was used to capture HSA (Table 1, regions 17, 21, 25, and 28), we also observed the presence of protein shroom, which is also associated with the actin protein. We believe the presence of this protein was linked with the antibody conjugate.

Furthermore, MP-PPEGMA-Ab was also incubated in different concentrations of human reference serum, followed by washing steps to remove any unbound proteins, and then loaded into the gel. Here, two distinct bands were observed (Figure S5, lanes 6–10), which correspond to both HSA (Table 1, regions 16, 20, 24, and 27) and Ab (Table 1, regions 17, 21, 25, and 28). As each lane was loaded with MP-PPEGMA-Ab, which had been incubated with human reference serum and subsequently washed, we concluded that the HSA present was conjugated with MP-PPEGMA-Ab, with the antibody fragments appearing as the secondary band at 55 kDa. Since no protein bands except for HSA and Ab were observed in the eluate from MP-PPEGMA-Ab and most of the proteins identified by LC-MS analysis were HSA, we concluded that nonspecific adsorption is efficiently suppressed by the CPB layer.

Additionally, no cross-reactivity was observed with our MP-PPEGMA-Ab against other albumins (Figure S6). Fetal bovine serum (FBS) was selected as it was a good candidate, mainly due to the presence of bovine serum albumin (BSA), which has a molecular size similar to that of HSA (67 kDa), while containing other proteins that may nonspecifically adsorb to the surface of the particles. As expected, we did not observe any cross-reactivity of our particles in the FBS solution, as we modified our particles with antibodies to specifically capture HSA (Figure S6, lane 4). Additionally, we found that there was no evidence of nonspecific protein fouling on the particle surface (Figure S7). Interestingly, when we incubated our magnetic particles in 0.1% (v/v) HSA in FBS, we found evidence of nonspecific protein absorption to our particles modified with BPE (Figure S7, lane 1). However, once we modified the surface with PPEGMA, we saw a large reduction in protein adsorption, due to the hydrophilic nature of PPEGMA, where the presence of the polymer brush structure minimized nonspecific adsorption. Moreover, following the modification of these polymer brushes with an anti-HSA antibody, we saw the presence of our proteins via SDS–PAGE, where there was a visible band present (Figures S7 and S8).

### 3.3 Indirect and Direct Quantification of Human Serum Albumin on MP-PPEGMA-Ab

To confirm the capture efficiency of our MP-PPEGMA-Ab system toward the targeted analyte, we performed an ELISA on both the solution supernatant (indirect) and our particle scaffold (direct) (Scheme 2; the red dotted circle indicates indirect analysis, and the green one direct analysis). First, we measured the protein content of the supernatant (indirect), where we determined the amount of protein captured on the surface of our particles by a simple mass balance (Figure 2A). It has been previously reported that the presence of nanoparticles in solutions can cause light scattering artifacts that can affect the absorption measurement.^70^ By incubating our particles with human serum containing known concentrations of HSA, we can separate captured HSA from the solution phase by using a simple magnetic force. Subsequently, this allowsus to quantify any remaining HSA present within the serum solution, where we indirectly quantify the amount of HSA captured by our particle system. After adding our antibody-modified CPB magnetic particles to human serum with varying concentrations of HSA, ranging from 4 to 86 ng mL^–1^ for one hour, subsequent ELISAs revealed that all residual supernatant solutions contained concentrations of HSA that were lower than the working detection limit of the assay (<3.1 ng mL^–1^).

**Figure 2:**
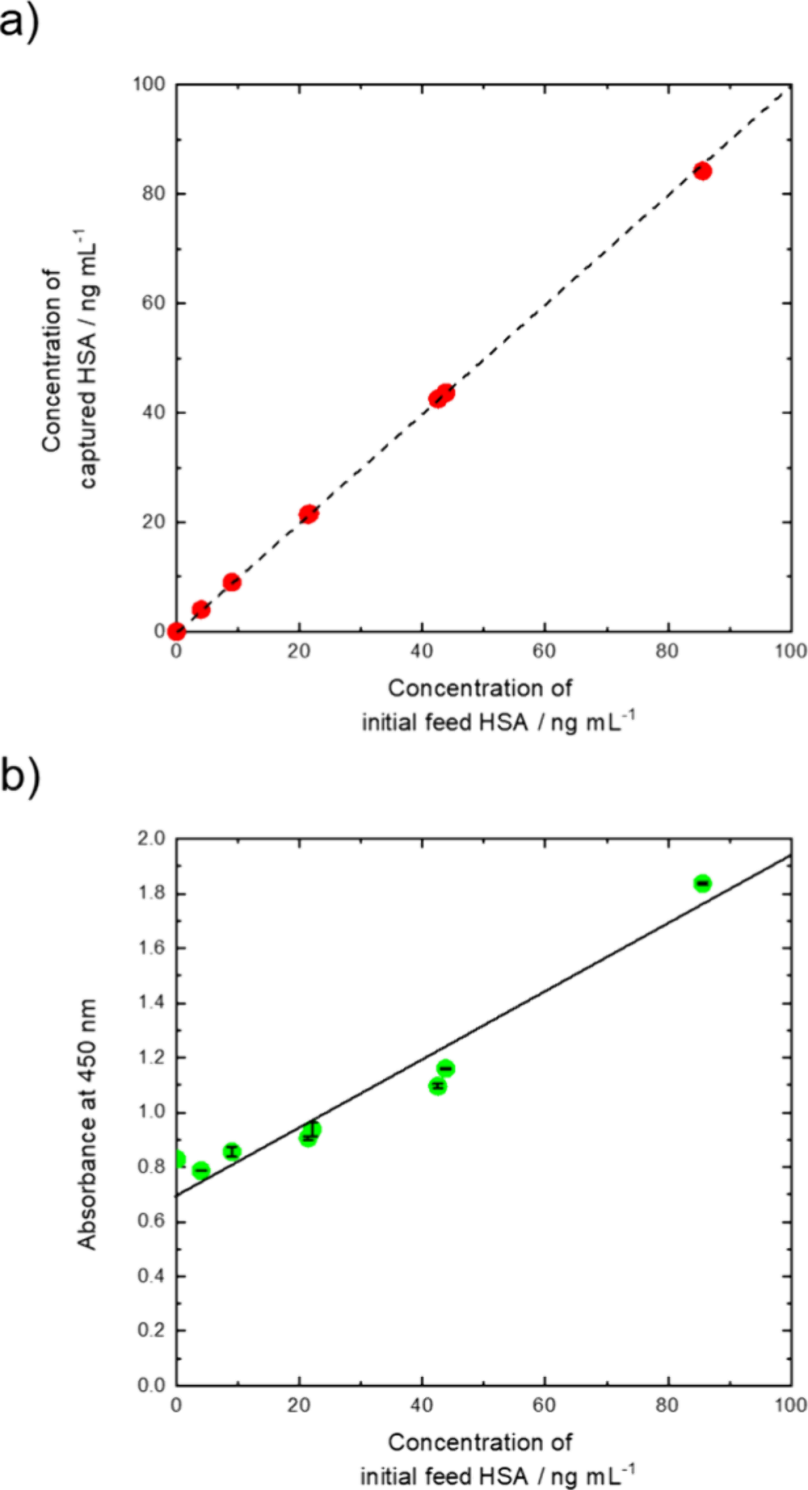
Human serum albumin capture efficiency using MP-PPEGMA-Ab in human serum, measured by (a) an indirect ELISA or (b) a direct ELISA. (a) ELISA on the supernatant following incubation with MP-PPEGMA-Ab and (b) sandwich ELISA using MP-PPEGMA-Ab as the substrate. The best fit line was determined to be absorbance = 0.012­[HSA] + 0.71.

In other words, more than 96% of the HSA present in the serum was bound to MP-PPEGMA-Ab. As shown in Figure 2a, we saw a linear relationship between the concentration of HSA initially in solution and HSA captured on the surface, where the capture efficiency was ∼100%. While we only tested samples containing less than 100 ng mL^–1^, it is possible that the dynamic range of our platform is greater than 100 ng mL^–1^ as the surface may not be fully saturated with HSA, where the theoretical saturation limit of our modified particles was determined to be approximately 1300 μg mL^–1^, as shown in Table S4. This is likely due to the orientation and structure of the PPEGMA CPB structure, resulting in a high concentration of Ab located on the surface. However, it should be noted that the actual saturation limit would be lower than the theoretical value as the orientation or structure of the antibody may change during the conjugation step, and further testing would be needed to confirm the true dynamic range of the sensor.

Because of the high capture efficiency of our particles, we then used our MP-PPEGMA-Ab as a platform for a sandwich ELISA (direct). Unlike conventional sandwich ELISAs, which involve multiple laborious steps, such as surface modification and blocking, these particles can be used directly as the ELISA platform, thus simplifying the process (Figure S9).

By incubating our MP-PPEGMA-Ab with different concentrations of human reference serum containing known concentrations of HSA and applying an external magnetic force, we could concentrate HSA while separating all unbound proteins in the serum. Then by incubating these particles with an HRP-conjugated antibody and performing a sandwich ELISA, we could directly measure the antigen concentration using TMB. As shown in Figure 2b, we would detect varying antigen concentrations, where we then calculated the limit of detection (LOD). As the limit of detection was defined as the mean value of the background measurements plus three times the standard deviation, resulting in an LOD of 10 ng mL^–1^.

### 3.4 Indirect and Direct Quantification of HSA in Whole Human Plasma on MP-PPEGMA-Ab

As the CPBs synthesized onto our MPs showed excellent antifouling behavior toward nonspecific adsorption, we tested our diagnostic assay in human plasma. Human plasma is a complex biological fluid that contains a multitude of different components, such as proteins, such as fibrinogen and albumin, and immunoglobulins.^71^ Because of this, being able to detect key analytes in serum has been reported to be difficult without sample purification, where nonspecific absorption has been reported to affect the sensitivity and functionality of biosensors in the past.^72–74^

We first confirmed whether the MP-PPEGMA-Ab system could be used to capture HSA in whole human plasma by an indirect ELISA. Here, we incubated our particles in different concentrations of human whole plasma, whereby the concentration of HSA was quantified prior to use (65 mg mL^–1^, as described in the Materials and Methods). By incubating our particles for one hour, we measured the supernatant solution to determine the amount of uncaptured HSA present in the remaining supernatant solution. As shown in Figure 3a, we found that our MP-PPEGMA-Ab could reliably capture HSA in unpurified plasma, where there was no difference in the trends between HSA in a single solution, or within the plasma/complex system. This suggests that the presence of the concentrated polymer brush structure minimized nonspecific protein absorption, allowing for a higher analyte sensitivity. We then measured the concentration of HSA on the surface of our MP-PEGMA-Ab system with a modified sandwich ELISA (Figure 3b; see Figure S9 for more details). Here, by using the magnetic particles as the platform for the assay, we incubated the particles with an HRP-modified antibody, whereby the concentration of HSA on the surface was determined using TMB. Here we found that the absorbance linearly decreased with decreasing plasma concentration. This indicates that MP-PEGMA-Ab could accurately determine the concentration of HSA in whole human plasma, where the presence of the CPB structure would minimize nonspecific protein interactions on the surface, allowing for a higher sensor sensitivity. Subsequent calculations revealed that the LOD for HSA in whole human plasma was 6.4 ng mL^–1^, which is comparable to or better than those from recent HSA detection studies, where concentrations in the milligram per milliliter range have been cited.^75–77^ To further support this observation, we used bare organosilica-modified MPs without the PPEGMA brushes (pristine MP), incubated them with human plasma, and quantified the amount of adsorbed HSA by an ELISA (Figure 3c). We found that the absorbance level of the pristine MPs was approximately 30% lower than that of the CPB-modified counterpart (MP-PPEGMA-Ab), indicating that a smaller amount of HSA had been captured on the particle surface. Without PPEGMA, nontargeted proteins in whole plasma can be adsorbed to the surface, which hindered the interaction between HSA and its complementary antibody due to steric hindrance. As PPEGMA forms a hydrophilic coating, with the configuration of the CPB structure, we were able to minimize nonspecific protein interactions on the surface to increase the sensitivity of our immunoassay. While it was observed that there was a stark difference between both pristine MPs and MP-PPEGMA-Ab, further statistical analysis to validate the sensitivity differences was not performed due to the limited sample size (*n* = 2). As it has been well documented that the presence of hydrophilic CPB coatings reduces nonspecific protein adsorption, we believe that these noticeable differences were due to the presence of the PPEGMA coating. Therefore, due to the enhanced sensitivity imparted by the presence of the CPB, the utility of these particles could be used for detection of other clinical applications in which low-abundance proteins can be easily detected without sample purification or pretreatment.^78,79^

**Figure 3:**
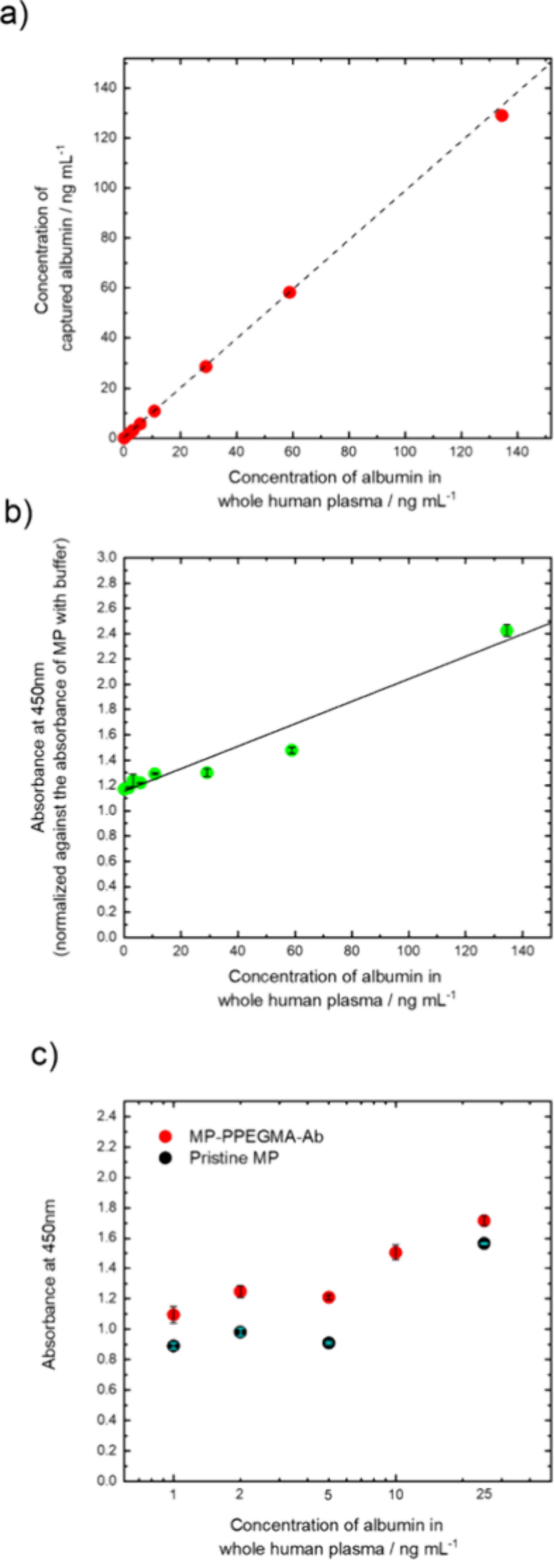
Human serum albumin capture efficiency using MP-PPEGMA-Ab in a whole human plasma solution measured by (a) an indirect ELISA or (b) a direct ELISA. (a) ELISA of the supernatant following incubation with MP-PPEGMA-Ab. (b) Sandwich ELISA using MP-PPEGMA-Ab as the substrate, compared to a whole human plasma solution. A best fit line was calculated to be absorbance = 0.0088­[HSA] + 1.15. (c) Comparison of unmodified MP particles (Pristine MP) and MP-PPEGMA-Ab. Experiments were performed in duplicate (*n* = 2). HSA concentrations in serum were 1, 2, 5, 10, and 25 ng mL^–1^.

## 4 Conclusion

In this work, we designed and developed a highly sensitive magnetic particle system for the capture and quantification of diagnostic marker proteins in clinical samples, such as serum or plasma. By using SI-ATRP, we were able to successfully graft concentrated PPEGMA brushes onto the surface of these particles to minimize nonspecific protein absorption. Subsequently, we then modified these particles with a targeting antibody to specifically target HSA, where we were able to achieve a limit of detection as low as 6.4 ng mL^–1^ in unpurified whole human plasma. From this, the use of CPBs showed excellent potential for the development of ultrasensitive diagnostic tools in applications in highly abundant protein environments.

## Supplementary Material



## Notes

Synthesis of PPEGMA-Br and PPEGMA-Azide, protein adsorption test comparing CPB and SDPB, material characterization of free PPEGMA-Br and MP-PPEGMA-Br, estimation of antibody functionalization, FTIR and ^1^H NMR analysis of modified PPEGMA, SDS–PAGE and protein staining of MP-PPEGMA-Ab in a protein solution, and schematic of ELISAs (PDF)

## References

[1] Chen Y.-T., Kolhatkar A. G., Zenasni O., Xu S., Lee T. R. (2017). Biosensing
Using Magnetic Particle Detection Techniques. Sensors.

[2] Avasthi, A. ; Caro, C. ; Pozo-Torres, E. ; Leal, M. P. ; García-Martín, M. L. Magnetic Nanoparticles as MRI Contrast Agents. In Surface-modified Nanobiomaterials for Electrochemical and Biomedicine Applications; Puente-Santiago, A. R. , Rodríguez-Padrón, D. , Eds.; Springer International Publishing: Cham, Switzerland, 2020; pp 49–91. 10.1007/978-3-030-55502-3_3

[3] Yan G.-P., Robinson L., Hogg P. (2007). Magnetic Resonance
Imaging Contrast
Agents: Overview and Perspectives. Radiography.

[4] Bañobre-López M., Teijeiro A., Rivas J. (2013). Magnetic Nanoparticle-Based Hyperthermia
for Cancer Treatment. Reports of Practical Oncology
& Radiotherapy.

[5] Hergt R., Dutz S., Müller R., Zeisberger M. (2006). Magnetic Particle
Hyperthermia: Nanoparticle Magnetism and Materials Development for
Cancer Therapy. J. Phys.: Condens. Matter.

[6] Kobayashi T. (2011). Cancer Hyperthermia
Using Magnetic Nanoparticles. Biotechnology
Journal.

[7] Plouffe B. D., Murthy S. K., Lewis L. H. (2015). Fundamentals and Application of Magnetic
Particles in Cell Isolation and Enrichment: A Review. Rep. Prog. Phys..

[8] Liu S., Li Z., Yu B., Wang S., Shen Y., Cong H. (2020). Recent Advances
on Protein Separation and Purification Methods. Adv. Colloid Interface Sci..

[9] Du M., Hou Z., Liu L., Xuan Y., Chen X., Fan L., Li Z., Xu B. (2022). Progress, Applications, Challenges and Prospects of
Protein Purification Technology. Front. Bioeng.
Biotechnol..

[10] Saxena A., Tripathi B. P., Kumar M., Shahi V. K. (2009). Membrane-Based
Techniques
for the Separation and Purification of Proteins: An Overview. Adv. Colloid Interface Sci..

[11] Schwalbe M., Pachmann K., Höffken K., Clement J. H. (2006). Improvement of the
Separation of Tumour Cells from Peripheral Blood Cells Using Magnetic
Nanoparticles. J. Phys.: Condens. Matter.

[12] Xu H., Aguilar Z. P., Yang L., Kuang M., Duan H., Xiong Y., Wei H., Wang A. (2011). Antibody Conjugated
Magnetic Iron Oxide Nanoparticles for Cancer Cell Separation in Fresh
Whole Blood. Biomaterials.

[13] Chou T.-C., Hsu W., Wang C.-H., Chen Y.-J., Fang J.-M. (2011). Rapid and Specific
Influenza Virus Detection by Functionalized Magnetic Nanoparticles
and Mass Spectrometry. J. Nanobiotechnol.

[14] Jat S. K., Gandhi H. A., Bhattacharya J., Sharma M. K. (2021). Magnetic Nanoparticles:
An Emerging Nano-Based Tool to Fight against Viral Infections. Mater. Adv..

[15] Chen G. D., Alberts C. J., Rodriguez W., Toner M. (2010). Concentration and Purification
of Human Immunodeficiency Virus Type 1 Virions by Microfluidic Separation
of Superparamagnetic Nanoparticles. Anal. Chem..

[16] Robinson K.
J., Coffey J. W., Muller D. A., Young P. R., Kendall M. A. F., Thurecht K. J., Grøndahl L., Corrie S. R. (2015). Comparison between
Polyethylene Glycol and Zwitterionic Polymers as Antifouling Coatings
on Wearable Devices for Selective Antigen Capture from Biological
Tissue. Biointerphases.

[17] Walker J. A., Robinson K. J., Munro C., Gengenbach T., Muller D. A., Young P. R., Lua L. H. L., Corrie S. R. (2019). Antibody-Binding,
Antifouling Surface Coatings Based on Recombinant Expression of Zwitterionic
EK Peptides. Langmuir.

[18] Lowe S., O’Brien-Simpson N. M., Connal L. A. (2015). Antibiofouling Polymer
Interfaces: Poly­(Ethylene Glycol) and Other Promising Candidates. Polym. Chem..

[19] Zhao Y., Gao N., Feng Y., Shi H., Sun W., Shen K., Wang Y., Shi S., Gong Y. (2018). Fabrication of Robust
Biomimetic Coating by Integrated Physisorption/Chemical Crosslinking
Technique. Appl. Surf. Sci..

[20] Charnley M., Textor M., Acikgoz C. (2011). Designed Polymer
Structures with
Antifouling-Antimicrobial Properties. React.
Funct. Polym..

[21] Yu L., Hou Y., Cheng C., Schlaich C., Noeske P.-L. M., Wei Q., Haag R. (2017). High-Antifouling
Polymer Brush Coatings on Nonpolar Surfaces via
Adsorption-Cross-Linking Strategy. ACS Appl.
Mater. Interfaces.

[22] Jeon S. I., Lee J. H., Andrade J. D., De Gennes P. G. (1991). Protein—Surface
Interactions in the Presence of Polyethylene Oxide: I. Simplified
Theory. J. Colloid Interface Sci..

[23] Jeon S. I., Andrade J. D. (1991). Protein—Surface Interactions in the Presence
of Polyethylene Oxide: II. Effect of Protein Size. J. Colloid Interface Sci..

[24] Al-Ani A., Pingle H., P Reynolds N., Wang P.-Y., Kingshott P. (2017). Tuning the
Density of Poly­(Ethylene Glycol) Chains to Control Mammalian Cell
and Bacterial Attachment. Polymers.

[25] Kingshott P., Thissen H., Griesser H. J. (2002). Effects
of Cloud-Point Grafting,
Chain Length, and Density of PEG Layers on Competitive Adsorption
of Ocular Proteins. Biomaterials.

[26] Perry J. L., Reuter K. G., Kai M. P., Herlihy K. P., Jones S. W., Luft J. C., Napier M., Bear J. E., DeSimone J. M. (2012). PEGylated
PRINT Nanoparticles: The Impact of PEG Density on Protein Binding,
Macrophage Association, Biodistribution, and Pharmacokinetics. Nano Lett..

[27] Yang Q., Jones S. W., Parker C. L., Zamboni W. C., Bear J. E., Lai S. K. (2014). Evading Immune Cell
Uptake and Clearance Requires PEG
Grafting at Densities Substantially Exceeding the Minimum for Brush
Conformation. Mol. Pharmaceutics.

[28] Dubey R., Shende P. (2024). Potential of Brush
and Mushroom Conformations in Biomedical
Applications. Chem. Pap..

[29] Li M., Jiang S., Simon J., Paßlick D., Frey M.-L., Wagner M., Mailänder V., Crespy D., Landfester K. (2021). Brush Conformation of Polyethylene
Glycol Determines the Stealth Effect of Nanocarriers in the Low Protein
Adsorption Regime. Nano Lett..

[30] Ding Z., Chen C., Yu Y., de Beer S. (2022). Synthetic Strategies
to Enhance the Long-Term Stability of Polymer Brush Coatings. J. Mater. Chem. B.

[31] Zhang X., Zhang J., Dong L., Ren S., Wu Q., Lei T. (2017). Thermoresponsive Poly­(Poly­(Ethylene
Glycol) Methylacrylate)­s Grafted
Cellulose Nanocrystals through SI-ATRP Polymerization. Cellulose.

[32] Xiu K. M., Cai Q., Li J. S., Yang X. P., Yang W. T., Xu F. J. (2012). Anti-Fouling
Surfaces by Combined Molecular Self-Assembly and Surface-Initiated
ATRP for Micropatterning Active Proteins. Colloids
Surf., B.

[33] Jiang S., Cao Z. (2010). Ultralow-Fouling, Functionalizable, and Hydrolyzable Zwitterionic
Materials and Their Derivatives for Biological Applications. Adv. Mater..

[34] Ladd J., Zhang Z., Chen S., Hower J. C., Jiang S. (2008). Zwitterionic
Polymers Exhibiting High Resistance to Nonspecific Protein Adsorption
from Human Serum and Plasma. Biomacromolecules.

[35] Zhang Y., Li M., Li B., Sheng W. (2024). Surface Functionalization with Polymer
Brushes via Surface-Initiated Atom Transfer Radical Polymerization:
Synthesis, Applications, and Current Challenges. Langmuir.

[36] Huang C.-J., Brault N. D., Li Y., Yu Q., Jiang S. (2012). Controlled
Hierarchical Architecture in Surface-Initiated Zwitterionic Polymer
Brushes with Structurally Regulated Functionalities. Adv. Mater..

[37] Yoshikawa C., Qiu J., Shimizu Y., Huang C.-F., Gelling O.-J., van den
Bosch E. (2017). Concentrated Polymer Brush-Modified Silica Particle Coating Confers
Biofouling-Resistance on Modified Materials. Materials Science and Engineering: C.

[38] Yoshikawa C., Goto A., Tsujii Y., Fukuda T., Yamamoto K., Kishida A. (2005). Fabrication of High-Density
Polymer Brush on Polymer
Substrate by Surface-Initiated Living Radical Polymerization. Macromolecules.

[39] Jian M., Sun X., Zhang H., Li X., Li S., Wang Z. (2024). Development
of a Peptide Microarray-Based Metal-Enhanced Fluorescence Assay for
Ultrasensitive Detection of Multiple Matrix Metalloproteinase Activities
by Using a Gold Nanorod-Polymer Substrate. Biosens.
Bioelectron..

[40] Yuan L., Wei W., Liu S. (2012). Label-Free
Electrochemical Immunosensors Based on Surface-Initiated
Atom Radical Polymerization. Biosens. Bioelectron..

[41] Ohno K., Morinaga T., Koh K., Tsujii Y., Fukuda T. (2005). Synthesis
of Monodisperse Silica Particles Coated with Well-Defined, High-Density
Polymer Brushes by Surface-Initiated Atom Transfer Radical Polymerization. Macromolecules.

[42] Yoshikawa C., Hattori S., Huang C.-F., Kobayashi H., Tanaka M. (2021). In Vitro and in Vivo Blood Compatibility
of Concentrated
Polymer Brushes. J. Mater. Chem. B.

[43] Zhao X., Wang N., Chen H., Bai L., Xu H., Wang W., Yang H., Wei D., Yang L., Cheng Z. (2020). Preparation of a Novel Sandwich-Type
Electrochemical Immunosensor
for AFP Detection Based on an ATRP and Click Chemistry Technique. Polym. Chem..

[44] Tsujii, Y. ; Ejaz, M. ; Yamamoto, S. ; Ohno, K. ; Urayama, K. ; Fukuda, T. Structure and Properties of High-Density Polymer Brushes. In Polymer Brushes; John Wiley & Sons, Ltd., 2004; pp 273–286. 10.1002/3527603824.ch14

[45] Yoshikawa C., Goto A., Tsujii Y., Ishizuka N., Nakanishi K., Fukuda T. (2007). Surface Interaction of Well-Defined, Concentrated Poly­(2-Hydroxyethyl
Methacrylate) Brushes with Proteins. J. Polym.
Sci., Part A: Polym. Chem..

[46] Xu C., Ohno K., Ladmiral V., Composto R. J. (2008). Dispersion of Polymer-Grafted
Magnetic Nanoparticles in Homopolymers and Block Copolymers. Polymer.

[47] Ohno K., Mori C., Akashi T., Yoshida S., Tago Y., Tsujii Y., Tabata Y. (2013). Fabrication
of Contrast Agents for
Magnetic Resonance Imaging from Polymer-Brush-Afforded Iron Oxide
Magnetic Nanoparticles Prepared by Surface-Initiated Living Radical
Polymerization. Biomacromolecules.

[48] Ohno K., Sakaue M., Mori C. (2018). Magnetically
Responsive Assemblies
of Polymer-Brush-Decorated Nanoparticle Clusters That Exhibit Structural
Color. Langmuir.

[49] Yoshikawa C., Qiu J., Huang C.-F., Shimizu Y., Suzuki J., van den
Bosch E. (2015). Non-Biofouling Property of Well-Defined Concentrated Polymer Brushes. Colloids Surf., B.

[50] Arizaga A., Millán A., Schubert U., Palacio F. (2013). Synthesis of Silica-Coated
Aqueous Ferrofluids through Ligand Exchange with a New Organosilica
Precursor. J. Mater. Sci..

[51] Tsujii, Y. ; Ohno, K. ; Yamamoto, S. ; Goto, A. ; Fukuda, T. Structure and Properties of High-Density Polymer Brushes Prepared by Surface-Initiated Living Radical Polymerization. In Surface-Initiated Polymerization I; Jordan, R. , Ed.; Springer: Berlin, 2006; pp 1–45. 10.1007/12_063

[52] Husseman M., Malmström E. E., McNamara M., Mate M., Mecerreyes D., Benoit D. G., Hedrick J. L., Mansky P., Huang E., Russell T. P., Hawker C. J. (1999). Controlled Synthesis of Polymer Brushes
by “Living” Free Radical Polymerization Techniques. Macromolecules.

[53] Zhang H., Jiang Y., Yu Q. (2010). Grafting Polymer
Brushes from Glass
Fibers by Surface-Initiated ATRP. Macromol.
React. Eng..

[54] Pyun J., Jia S., Kowalewski T., Patterson G. D., Matyjaszewski K. (2003). Synthesis
and Characterization of Organic/Inorganic Hybrid Nanoparticles: Kinetics
of Surface-Initiated Atom Transfer Radical Polymerization and Morphology
of Hybrid Nanoparticle Ultrathin Films. Macromolecules.

[55] von
Werne T., Patten T. E. (1999). Preparation of Structurally Well-Defined
Polymer-Nanoparticle Hybrids with Controlled/Living Radical Polymerizations. J. Am. Chem. Soc..

[56] Saeed A. O., Magnusson J. P., Moradi E., Soliman M., Wang W., Stolnik S., Thurecht K. J., Howdle S. M., Alexander C. (2011). Modular Construction
of Multifunctional Bioresponsive Cell-Targeted Nanoparticles for Gene
Delivery. Bioconjugate Chem..

[57] Sakakibara K., Nishiumi K., Shimoaka T., Hasegawa T., Tsujii Y. (2019). pMAIRS Analysis
on Chain-End Functionalization of Densely Grafted, Concentrated Polymer
Brushes. Macromolecules.

[58] Geng J., Lindqvist J., Mantovani G., Haddleton D. M. (2008). Simultaneous
Copper­(I)-Catalyzed Azide-Alkyne Cycloaddition (CuAAC) and Living
Radical Polymerization. Angew. Chem., Int. Ed..

[59] Arques S. (2018). Human Serum
Albumin in Cardiovascular Diseases. European
Journal of Internal Medicine.

[60] Manolis A. A., Manolis T. A., Melita H., Mikhailidis D. P., Manolis A. S. (2022). Low Serum Albumin: A Neglected Predictor in Patients
with Cardiovascular Disease. European Journal
of Internal Medicine.

[61] Zhang J., Zhang R., Wang Y., Li H., Han Q., Wu Y., Wang T., Liu F. (2019). The Level of Serum
Albumin Is Associated
with Renal Prognosis in Patients with Diabetic Nephropathy. J. Diabetes Res..

[62] Lee C., Le M.-P., Wallingford J. B. (2009). The Shroom Family Proteins Play Broad
Roles in the Morphogenesis of Thickened Epithelial Sheets. Dev. Dyn..

[63] Liu W., Xiu L., Zhou M., Li T., Jiang N., Wan Y., Qiu C., Li J., Hu W., Zhang W., Wu J. (2024). The Critical
Role of the Shroom Family Proteins in Morphogenesis, Organogenesis
and Disease. Phenomics.

[64] Durbin M. D., O’Kane J., Lorentz S., Firulli A. B., Ware S. M. (2020). SHROOM3
Is Downstream of the Planar Cell Polarity Pathway and Loss-of-Function
Results in Congenital Heart Defects. Dev. Biol..

[65] Janmey P. A., Lind S. E. (1987). Capacity of Human Serum to Depolymerize Actin Filaments. Blood.

[66] Manning J. M., Dumoulin A., Manning L. R., Chen W., César
Padovan J., Chait B. T., Popowicz A. (1999). Remote Contributions
to Subunit Interactions: Lessons from Adult and Fetal Hemoglobins. Trends Biochem. Sci..

[67] Churg A., Dai J., Zay K., Karsan A., Hendricks R., Yee C., Martin R., MacKenzie R., Xie C., Zhang L., Shapiro S., Wright J. L. (2001). Alpha-1-Antitrypsin and a Broad Spectrum
Metalloprotease Inhibitor, RS113456, Have Similar Acute Anti-Inflammatory
Effects. Laboratory Investigation.

[68] O’Brien M. E., Murray G., Gogoi D., Yusuf A., McCarthy C., Wormald M. R., Casey M., Gabillard-Lefort C., McElvaney N. G., Reeves E. P. (2022). A Review of Alpha-1 Antitrypsin Binding
Partners for Immune Regulation and Potential Therapeutic Application. Int. J. Mol. Sci..

[69] Gardel M. L., Schneider I. C., Aratyn-Schaus Y., Waterman C. M. (2010). Mechanical Integration
of Actin and Adhesion Dynamics in Cell Migration. Annu. Rev. Cell Dev. Biol..

[70] Wang J., Xu C., Nilsson A. M., Fernandes D. L. A., Strömberg M., Wang J., Niklasson G. A. (2019). General
Method for Determining Light
Scattering and Absorption of Nanoparticle Composites. Advanced Optical Materials.

[71] Anderson N. L., Anderson N. G. (2002). The Human Plasma
Proteome: History, Character, and
Diagnostic Prospects *. Molecular & Cellular
Proteomics.

[72] Ahluwalia A., Giusto G., De Rossi D. (1995). Non-Specific Adsorption
on Antibody
Surfaces for Immunosensing. Materials Science
and Engineering: C.

[73] Lichtenberg J. Y., Ling Y., Kim S. (2019). Non-Specific Adsorption
Reduction
Methods in Biosensing. Sensors.

[74] Frutiger A., Tanno A., Hwu S., Tiefenauer R. F., Vörös J., Nakatsuka N. (2021). Nonspecific
Binding—Fundamental
Concepts and Consequences for Biosensing Applications. Chem. Rev..

[75] Hu Q., Iwanaga M., Tang Y. (2024). Metasurface
Platform Incorporating
Aggregation Induced Emission Based Biosensor for Enhanced Human Serum
Albumin Detection. Advanced Optical Materials.

[76] Jangannanavar V. D., Basavanagoudra H., Matteppanavar S., Vaddar H., Nagarajappa H., Patil M. K., Inamdar S. R., Goudar K. M. (2025). Honey-Mediated CeO_2_ Nanoparticles: A Cost-Effective Approach for Electrochemical
Biosensing of Human Serum Albumin. Langmuir.

[77] Thongwattana T., Chaiyo R., Ponsanti K., Tangnorawich B., Pratumpong P., Toommee S., Jenjob R., Yang S.-G., Parcharoen Y., Natphopsuk S., Pechyen C. (2024). Synthesis of Silver
Nanoparticles and Gold Nanoparticles Used as Biosensors for the Detection
of Human Serum Albumin-Diagnosed Kidney Disease. Pharmaceuticals.

[78] Boschetti E., Righetti P. G. (2023). Low-Abundance Protein Enrichment for Medical Applications:
The Involvement of Combinatorial Peptide Library Technique. International Journal of Molecular Sciences.

[79] Millioni R., Tolin S., Puricelli L., Sbrignadello S., Fadini G. P., Tessari P., Arrigoni G. (2011). High Abundance
Proteins
Depletion vs Low Abundance Proteins Enrichment: Comparison of Methods
to Reduce the Plasma Proteome Complexity. PLoS
One.

