# Supplementary material for: Concentrated Polymer Brush-Modified Magnetic Particles for a Diagnostic Immunoassay
Source: Langmuir. 2025 Nov 25;41(48):32432–42. doi: 10.1021/acs.langmuir.5c04175 (PMC12874528; doi:10.1021/acs.langmuir.5c04175)
Supplement: Supplementary file 1 [file la5c04175_si_001.pdf]

Supplementary Information

Concentrated Polymer Brush-Modified Magnetic Particles for a Diagnostic Immunoassay

Gabriel Tai Huynh[a], Jun Qiu[b], Edith van den Bosch[b], Tomohiko Yamazaki [a, c] and Chiaki Yoshikawa *[a, c]

[a]Research Center for Macromolecules and Biomaterials, National Institute for Materials Science (NIMS), 1-2-1 Sengen, Tsukuba, Ibaraki 305-0047, Japan

[b]DSM Ahead/TS, 6167 DR Geleen, The Netherlands

[c]Graduate School of Life Science, Hokkaido University, Kita 10, Nishi 8, Sapporo 060-0808, Japan

1. Synthesis of free poly[poly(ethylene glycol) methyl methacrylate bromide] (PPEGMA-Br)

Synthesis of PPEGMA-Br in solution was prepared as followed: N_2-purged methanol solution (6.60 g) of poly(ethylene glycol) methyl methacrylate (PEGMA, 6.60 g, 13.94 mmol), copper (I) bromide (Cu(I)Br, 10.0 mg, 0.070 mmol), 2-2'-Bipyridyl (Bpy, 21.78 mg, 0.139 mmol) and free initiator Ethyl-2-bromoisobutyrate (EBIB, 13.59 mg, 0.070 mmol) was prepared in an oxygen-free environment, and combined with a Schlenk tube and sealed with a three-way stopcock valve. The resulting solution was mixed in a shaking oil bath for 3.5 hours at 30 °C. After polymerization, the resultant polymer was precipitated five times using a solution of hexane and diethyl oxide (ratio 7:3 wt%) to remove unreacted monomer and other impurities. Subsequent analysis by gel permeation chromatography (GPC) and proton nuclear magnetic resonance (^{1}H NMR) confirmed the successful precipitation of the polymer.

Table S1. GPC analysis of the free PPEGMA-Br

	M_n[a]	PDI[a]	$M_{n,conv}$
**Before purification**	17900	1.1	7600

[a] Estimated by PMMA calibra

2. Terminal azidation of free PPEGMA in solution (PPEGMA-Azide).

Modification of the free PPEGMA-Br was as followed: PPEGMA-Br (500 mg) and sodium azide (NaN_3, 68.9 mg) were first dissolved in 11.00 g of dimethylformamide (DMF), before heating the mixture to 50°C for 24 hours. Afterwards, 20 mL of toluene was added to the solution and then the solution was centrifuge at 12,000 rpm for 10 minutes to remove excess salt. The solution was then purified by first evaporating toluene using a rotary evaporator, before removing excess DMF under vacuum drying. Subsequently, the sample was analyzed with ^{1}H NMR and ATR-IR to confirm the presence of the azido terminal group.

3. Protein adsorption on MP-PPEGMA-Br with different graft densities.

MPs-PPEHMA-Br with different graft densities (Table S3) were prepared according to our previous report[3]. MPs (5 mg) were dispersed in 100 μL of phosphate-buffered saline (PBS). Fetal bovine serum (FBS) (900 μL) was then added to the solution, and the mixture was incubated at 37 °C for 1 h. The MPs were washed five times with 1 mL 1X PBS to remove unbound protein. After washing, the MPs were dispersed in PBS to adjust the concentration to 0.25 mg mL^{-1}. Subsequently, 10 μL of the solution was loaded in a polyacrylamide gel, and electrophoresis was conducted according to the manufacturer provided protocol (NuPAGE®Bis-Tris Mini Gels, Life Technologies™). Protein bands were visualized by silver staining. Stained gel images were obtained using a scanner (CanonScan8800F, Canon, Japan).

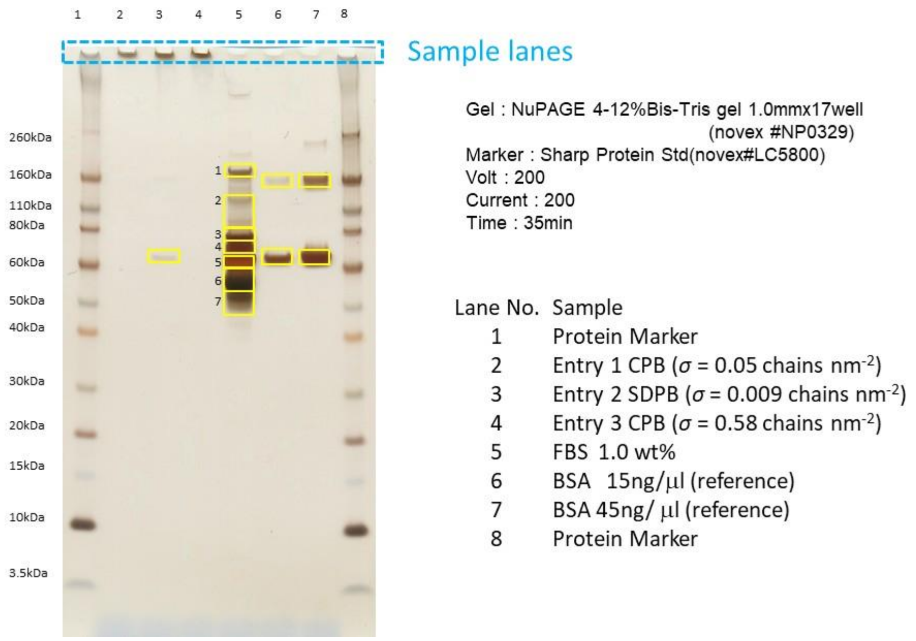

Figure S1. Protein bands visualized on a gel sheet. MPs with PPEGMA brushes were incubated in diluted FBS (1.0 wt%) for 1h at 37 °C.

Table S2. Characterization data of the PPEGMA coating on the magnetic particles (MP-PPEGMA-Br)

Entry	M_n[a]	M_w/M_n[a]	$M_{n,conv}$[b]	Graft amount[c] / g g^{-1}	σ / chains nm^{-2}	σ*
1	45200	1.2	53200	0.28	0.41	0.8
2	36500	1.2	38200	0.25	0.47	0.9
3	47200	1.2	48000	0.24	0.57	1.0

[a] Estimated by GPC-calibrated PMMA standards from free polymer in solution. [b]Determined by ^{1}H NMR spectroscopy from free polymer in solution. [c]Determined by TG-DTA using MP-PPEGMA-Br.

Table S3. MP-PPEGMA-Br with different graft densities used for the FBS protein adsorption test.

Entry	Brush type	$M_{n,conv}$	σ / chains nm^{-2}	σ*
1	CPB	49000	0.05	0.16
2	SDPB	43500	0.009	0.03
3	CPB	52300	0.58	1.0

Table S4. The amount of antibody on the graft polymers.

Entry	Pristine Ab[a] (mg mL^{-1})	Unreacted Ab[a] (mg mL^{-1})	Immobilized Ab[b] (mg mL^{-1})	The immobilized Ab[c] (nmol mg^{-1})	The graft chains[d] (nmol mg^{-1})	The molar ratio of Ab to the graft chains.
1	1.45	0.028	1.42	0.38	0.44	0.86
2	1.36	0.185	1.17	0.32	0.44	0.73

[a]Determined by BCA assay. [b][Immobilized Ab] = [Pristine Ab]-[Unreacted Ab]. [c]400 µl of antibody solution was used for the reaction. Molecular weight of Ab is 150K Da. [d]10mg of MP-PPEGMA-NHS was used for the reaction. The surface area of MPs is approx. 5.3x10^{18} nm^2/g and the graft density was approx. 0.5 chains/nm^2.

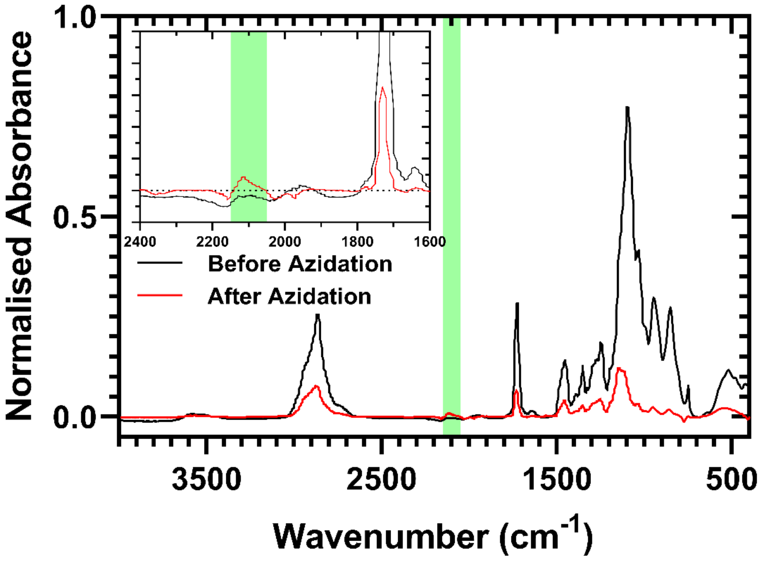

Figure S2. Fourier Transform Infrared Spectroscopy (FTIR) of free PPEGMA before (black) and after (red) azidation. Highlighted in green is the vibrational wavelength which corresponds to azides (-N_3) at 2100 cm^{-1}. Inset: Magnified section of the spectrum from 1600 cm^{-1} to 2400 cm^{-1}.

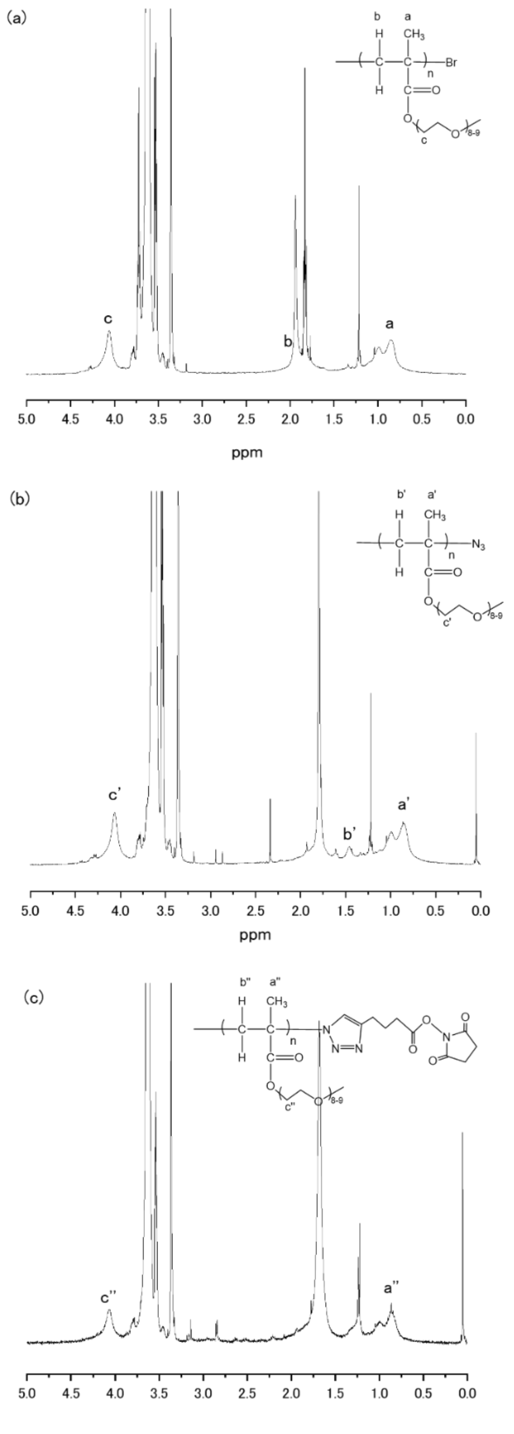

Figure S3. ^1H NMR of the free polymer, PPEGMA and subsequent modification: (a) ^1H NMR of PPEGMA before modification, (b) ^1H NMR of PPEGMA following azidation (PPEGMA-N_3), and (c), ^1H NMR of PPEGMA-N_3 following the azide-alkyne click reaction with N-(4-Pentynoyloxy) succinimide (PPEGMA-NHS).

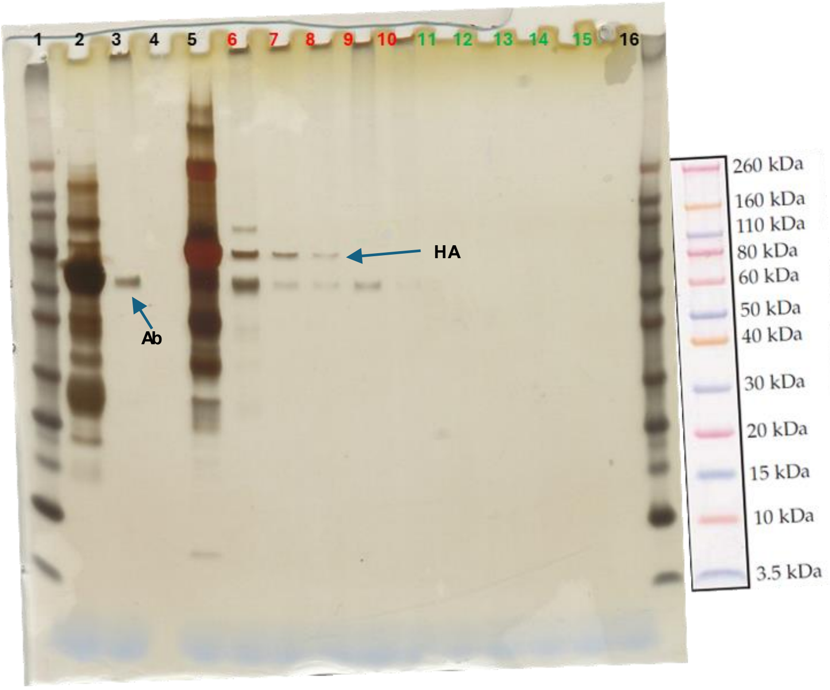

lane NO	Sample
1	Marker
2	Antibody (anti-HSA 1.0mg/ml)
3	MP-anitbody
4	blank
5	HSA 0.5 mg/ml
6	A (1.0 mg/ml HSA)
7	B (0.1 mg/ml HSA)
8	C (0.001 mg/ml HSA)
9	D (0.00001 mg/ml HSA)
10	E (0.0000001 mg/ml HSA)
11	Supernatant A
12	Supernatant B
13	Supernatant C
14	Supernatant D
15	Supernatant E
16	Marker

Figure S4. SDS-PAGE of MP-PPEGMA-Ab in different concentrations of HSA solution. MP-PPEGMA-Ab were incubated in different concentrations of HSA solution before diluting it to a final concentration of 0.25 mg/µL of MP-PPEGMA-Ab. Supernatant of the solution were ran concurrently to confirm the presence of uncaptured proteins on the surface.

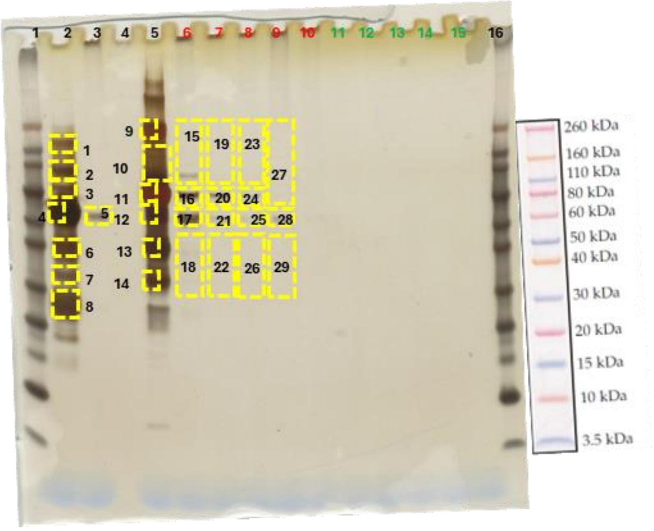

lane NO	Sample
1	Marker
2	Antibody (anti-HSA 1.0mg/ml)
3	MP-anitbody
4	blank
5	HSA 0.5 mg/ml
6	A (1.0 mg/ml HSA)
7	B (0.1 mg/ml HSA)
8	C (0.001 mg/ml HSA)
9	D (0.00001 mg/ml HSA)
10	E (0.0000001 mg/ml HSA)
11	Supernatant A
12	Supernatant B
13	Supernatant C
14	Supernatant D
15	Supernatant E
16	Marker

Figure S5. SDS-PAGE of MP-PPEGMA-Ab in different concentrations of HSA solution for LC-MS. MP-PPEGMA-Ab were incubated in different concentrations of HSA solution before diluting it to a final concentration of 0.25 mg/µL of MP-PPEGMA-Ab. Labels within the yellow dashed lines represent sections of the gel analysed in LC-MS.

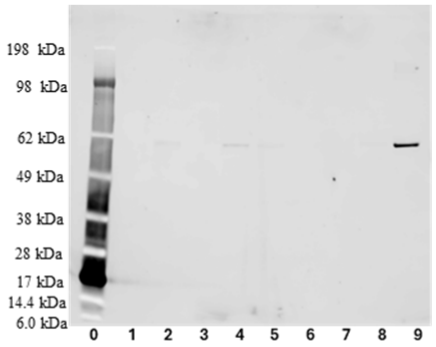

#	Sample Description:
0	Standard Ladder
1	MP-BPE: incubated **without** 0.1% v/v HSA in undiluted FBS
2	MP-PPEGMA-Br : incubated **without** 0.1% v/v HSA in undiluted FBS
3	MP-PPEGMA-NHS : incubated **without** 0.1% v/v HSA in undiluted FBS
4	MP-PPEGMA-Ab : incubated **without** 0.1% v/v HSA in undiluted FBS
5	MP-BPE: last fraction wash following incubated **with** 0.1% v/v HSA in undiluted FBS
6	MP-PPEGMA-Br : last fraction wash following incubated **with** 0.1% v/v HSA in undiluted FBS
7	MP-PPEGMA-NHS : last fraction wash following incubated **with** 0.1% v/v HSA in undiluted FBS
8	MP-PPEGMA-Ab : last fraction wash following incubated **with** 0.1% v/v HSA in undiluted FBS
9	0.1 % v/v HSA in undiluted FBS, then diluted 1:1000 in PBS

Figure S6. Protein staining through SYPRO Ruby Protein Stain on modified magnetic particles following incubation in FBS, where each modification step is shown in Figure 1. Lane 9 is a control to confirm that SYPRO Ruby Stain only stains proteins.

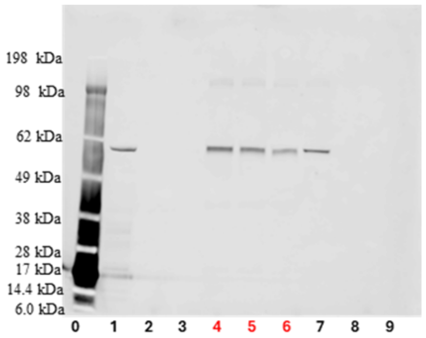

#	Sample Description:
0	Standard Ladder
1	MP-BPE: incubated **with** 0.1% v/v HSA in undiluted FBS, following six washing steps
2	MP-PPEGMA-Br : incubated **with** 0.1% v/v HSA in undiluted FBS, following six washing steps
3	MP-PPEGMA-NHS : incubated **with** 0.1% v/v HSA in undiluted FBS, following six washing steps
4	MP-PPEGMA-Ab : incubated **with** 0.1% v/v HSA in undiluted FBS, following six washing steps
5	MP-PPEGMA-Ab : incubated **with** 0.1% v/v HSA in undiluted FBS, following six washing steps
6	MP-PPEGMA-Ab : incubated **with** 0.1% v/v HSA in undiluted FBS, following six washing steps
7	0.1 % v/v HSA in undiluted FBS, then diluted 1:1000 in PBS
8	Anti-HSA antibody diluted 1:500 in PBS
9	Anti-HSA antibody diluted 1:100 in PBS

Figure S7. Protein staining through SYPRO Ruby Protein Stain on modified magnetic particles following incubation in 0.1% v/v HSA/FBS solution, following each modification step shown in Figure 1. Controls (Lane 7 to 9) showed confirmation that the stain selectively stain proteins and not antibodies.

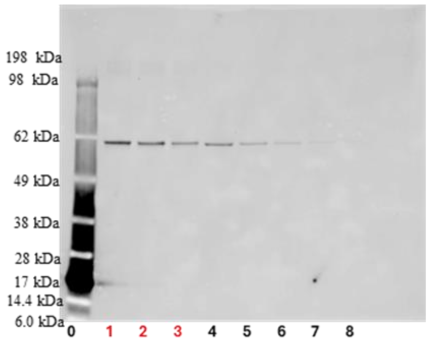

#	Sample Description:
0	Standard Ladder
1	MP-PPEGMA-Ab : incubated **with** 0.1% v/v HSA in undiluted FBS, following six washing steps
2	MP-PPEGMA-Ab : incubated **with** 0.1% v/v HSA in undiluted FBS, following six washing steps
3	MP-PPEGMA-Ab : incubated **with** 0.1% v/v HSA in undiluted FBS, following six washing steps
4	0.1 % v/v HSA in undiluted FBS, then diluted 1:1000 in PBS
5	0.1 % v/v HSA in undiluted FBS, then diluted 1:2500 in PBS
6	0.1 % v/v HSA in undiluted FBS, then diluted 1:5000 in PBS
7	0.1 % v/v HSA in undiluted FBS, then diluted 1:10000 in PBS
8	0.1 % v/v HSA in undiluted FBS, then diluted 1:25000 in PBS

Figure S8. Protein staining through SYPRO Ruby Protein Stain on MP-PPEGMA-Ab particles following incubation in 0.1% v/v HSA/FBS solution. Lane 4 to 8 are reference lanes to confirm the concentration of protein captured by the magnetic particles.

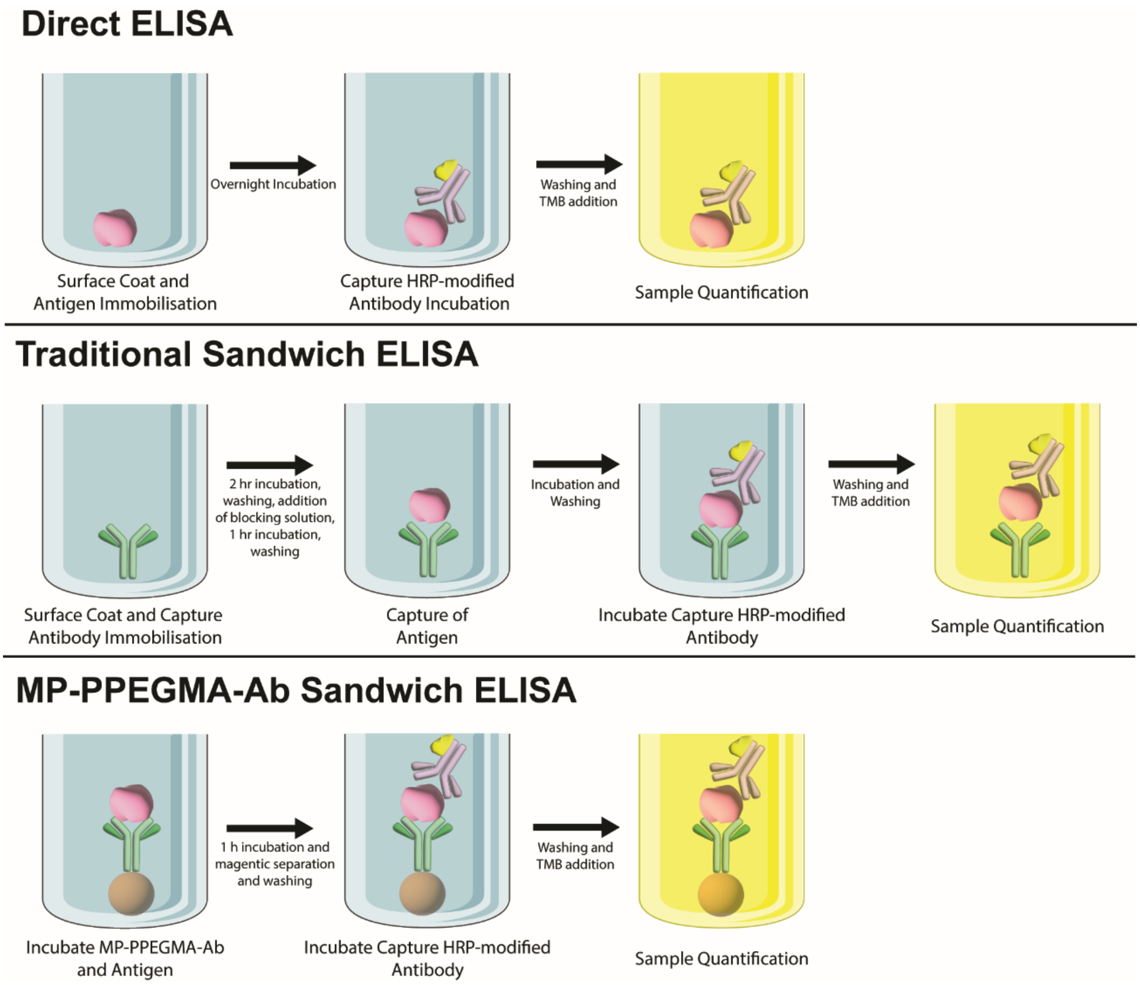

Figure S9. Schematic on the difference between i) Direct ELISA, ii) Traditional Sandwich ELISA, and iii) MP-PPEGMA-Ab Sandwich ELISA (current work)

Reference

(1) Oh, J. K.; Min, K.; Matyjaszewski, K. Preparation of Poly(Oligo(Ethylene Glycol) Monomethyl Ether Methacrylate) by Homogeneous Aqueous AGET ATRP. *Macromolecules* **2006**, *39* (9), 3161–3167. https://doi.org/10.1021/ma060258v.

(2) Hansen, N. M. L.; Haddleton, D. M.; Hvilsted, S. Fluorinated Bio-Acceptable Polymers via an ATRP Macroinitiator Approach. *Journal of Polymer Science Part A: Polymer Chemistry* **2007**, *45* (24), 5770–5780. https://doi.org/10.1002/pola.22326.

(3) Yoshikawa, C.; Hattori, S.; Huang, C.-F.; Kobayashi, H.; Tanaka, M. In Vitro and in Vivo Blood Compatibility of Concentrated Polymer Brushes. *J. Mater. Chem. B* **2021**, *9* (29), 5794–5804. https://doi.org/10.1039/D1TB00886B.
